# *MLH1*–93 G/a polymorphism is associated with *MLH1* promoter methylation and protein loss in dysplastic sessile serrated adenomas with *BRAF*^V600E^ mutation

**DOI:** 10.1186/s12885-017-3946-5

**Published:** 2018-01-05

**Authors:** Lochlan J. Fennell, Saara Jamieson, Diane McKeone, Tracie Corish, Megan Rohdmann, Tori Furner, Mark Bettington, Cheng Liu, Futoshi Kawamata, Catherine Bond, Jolieke Van De Pols, Barbara Leggett, Vicki Whitehall

**Affiliations:** 1 Conjoint Gastroenterology Laboratory, QIMR Berghofer Medical Research Institute, Brisbane, QLD Australia; 2 School of Health and Sport Science, University of the Sunshine Coast, Sunshine Coast, QLD Australia; 3 School of Medicine, University of Queensland, Brisbane, QLD Australia; 4 Envoi Specialist Pathology, Brisbane, QLD Australia; 5 Queensland University of Technology, Faculty of Health, Brisbane, QLD Australia; 6 Department of Gastroenterology and Hepatology, RBWH, Brisbane, QLD Australia; 7 Pathology Queensland, Brisbane, QLD Australia

**Keywords:** Colorectal cancer, BRAF, Mismatch repair, Sessile serrated adenoma, CpG Island Methylator phenotype

## Abstract

**Background:**

Sessile serrated adenomas with *BRAF* mutation progress rapidly to cancer following the development of dysplasia (SSAD). Approximately 75% of SSADs methylate the mismatch repair gene *MLH1*, develop mismatch repair deficiency and the resultant cancers have a good prognosis. The remaining SSADs and *BRAF* mutant traditional serrated adenomas (TSA) develop into microsatellite stable cancers with a poor prognosis. The reason for this dichotomy is unknown. In this study, we assessed the genotypic frequency of the *MLH1–*93 polymorphism rs1800734 in SSADs and TSAs to determine if the uncommon variant A allele predisposes to *MLH1* promoter hypermethylation.

**Methods:**

We performed genotyping for the *MLH1–*93 polymorphism, quantitative methylation specific PCR, and MLH1 immunohistochemistry on 124 SSAD, 128 TSA, 203 *BRAF* mutant CRCs and 147 control subjects with normal colonoscopy.

**Results:**

The minor A allele was significantly associated with a dose dependent increase in methylation at the *MLH1* promoter in SSADs (*p* = 0.022). The AA genotype was only observed in SSADs with MLH1 loss. The A allele was also overrepresented in BRAF mutant cancers with MLH1 loss. Only one of the TSAs showed loss of MLH1 and the overall genotype distribution in TSAs did not differ from controls.

**Conclusions:**

The *MLH1*–93 AA genotype is significantly associated with promoter hypermethylation and MLH1 loss in the context of SSADs. *BRAF* mutant microsatellite stable colorectal cancers with the AA genotype most likely arise in TSAs since the A allele does not predispose to methylation in this context.

## Background

Colorectal cancer is a heterogeneous disease that arises from a number of distinct molecular pathways [1]. The majority arise from conventional colorectal adenomas in which the initiating event is usually inactivation of the *APC* tumor suppressor gene [2, 3]. An important subgroup of colorectal cancers bear a mutation in the *BRAF* oncogene [4] and these cancers arise from serrated polyps initiated by the *BRAF* mutation [5]. There is a very strong association between *BRAF* mutation in colorectal cancer and aberrant DNA methylation of CpG islands which is associated with gene silencing when it occurs in promoter areas [6]. This has been described as the CpG Island Methylator Phenotype (CIMP) [7]. One of the important genes sometimes silenced by methylation is *MLH1* which encodes a mismatch repair protein. Loss of MLH1 expression results in mismatch repair deficiency and the rapid accumulation of mutations manifested as microsatellite instability (MSI) [8]. MSI cancers have a good prognosis but not all colorectal cancers with *BRAF* mutation and CIMP silence *MLH1* and those that remain microsatellite stable (MSS) have a particularly poor prognosis [9].

There are two types of serrated polyp from which *BRAF* mutant cancers arise. The most common is the sessile serrated adenoma which occurs predominantly in the proximal colon and in older women [1]. They are characterized by abnormal crypt architecture but do not have cytological dysplasia. They typically have both *BRAF* mutation and evolving CIMP but not MLH1 silencing or MSI. Development of cytological dysplasia in a sessile serrated adenoma (SSAD) is associated with rapid progression to invasive malignancy, it is at this stage that methylation-induced silencing of MLH1, and development of MSI may occur. These lesions ‘caught in the act’ of progressing to malignancy are rarely observed in the clinic, and account for approximately 1% of all sessile serrated adenomas. We have recently curated a series of dysplastic sessile serrated adenomas and shown that 75% of SSAD progress methylate MLH1, are MSI, and thus progress to *BRAF* mutant MSI cancers. For unknown reasons, 25% do not silence MLH1 and become *BRAF* mutant MSS cancers [10]. The second type of serrated polyp with malignant potential is the traditional serrated adenoma (TSA) which is an uncommon polyp occurring in the distal colon with an equal gender distribution [11]. *BRAF* mutation is present in 67% and the majority of these polyps show CIMP. They have a high malignant potential but even during malignant conversion silencing of MLH1 is extremely rare [11]. Thus TSAs are a source of *BRAF* mutant MSS cancers.

Whether the promoter of *MLH1* becomes sufficiently methylated to silence the gene in the setting of CIMP may not be a random, stochastic process. Several studies have associated a series of single nucleotide polymorphisms in the *MLH1* promoter with the occurrence of methylation-induced silencing in large series of cancers [12, 13]*.* The study by Mirakuya and colleagues found a significant association between *MLH1* methylation and the A allele of the rs1800734 single nucleotide polymorphism in a consecutive, unselected series of colorectal cancers, stratifying cancers into negative, partial or full methylation using bisulphite sequencing. Rs1800734 (or *MLH1*–93) is a polymorphism 93 base pairs from the *MLH1* translation start site. Subsequent studies have indicated a shift in protein binding as a result of this G > A polymorphism [13]. Further, a recent study by Liu et al. showed that the A allele was able to regulate an upstream gene, DCLK3, in a trans-acting manner [14]. They were unable to demonstrate a relationship between the polymorphism and methylation in vivo, but only MSS cell lines were studied [14]. The effect of the polymorphism on methylation may only occur in a particular cellular context.

We hypothesized that the A allele of *MLH1*–93 is an important factor influencing methylation-induced silencing of *MLH1* in the permissive environment of a *BRAF* mutant SSAD but not in the context of TSA.

## Methods

### Sample selection

Samples were obtained from Envoi Specialist Pathology (Envoi) Brisbane, Australia, over a six-year period and are part of two previously published series [10, 11]. Envoi Specialist Pathology is a community based specialist gastroenterology practice. These series include polyps and cancers removed both endoscopically and surgically. Tissue from Envoi was embedded in formalin fixed paraffin embedded (FFPE) blocks, with DNA extracted using chelex, as previously reported [15]. Cancers were obtained in a fresh state from patients undergoing surgery at the Royal Brisbane and Women’s Hospital, Brisbane, Australia, and from FFPE blocks at Envoi. Fresh samples were extracted using salt precipitation [16] and FFPE samples were extracted using chelex. For the control cohort, blood samples were taken from consenting patients who presented to gastroenterology clinics in Brisbane for investigation of symptoms and in whom subsequent colonoscopy showed no polyps or cancer.

### Pathological assessment

Each sample was review by independently by two expert pathologists. Criteria for the diagnosis of a traditional serrated adenoma can be found in Bettington et al., 2015 [11]. Criteria for the diagnosis of a dysplastic sessile serrated adenoma can be found in Bettington et al., 2017 [10].”

### BRAF and CIMP analysis

The BRAF V600E mutation was assessed in each sample using allelic discrimination as previously reported [11]. We assessed CIMP status using a methylation specific PCR with a marker panel consisting of *NEUROG1, SOCS1, CACNAIG, IGF2 and RUNX3* as reported by Weisenberger and colleagues [6]. To avoid the potential confounding of MLH1 loss secondary to Lynch Syndrome, only polyps and cancers bearing the *BRAF*^*V600E*^ mutation were included. BRAF mutation has previously been shown to be an excellent marker of somatic MLH1 loss due to promoter hypermethylatioon [17].

### MLH1 methylation and immunohistochemical analysis

For SSAD,TSA and cancer cohorts, *MLH1* methylation was determined by bisulfite conversion, followed by methylation specific qPCR as previously reported [10]. MLH1 protein expression was assessed by immunohistochemistry using previously reported methods [11], staining patterns were analyzed by an experienced gastrointestinal pathologist (MB).

### SNP genotyping analysis

*MLH1*–93 genotypes were determine by high resolution melt analysis using 2.4 mM MgCl_2_, 0.24 mM dNTP, 0.24uM forward primer (5-‘TGACTGGCATTCAAGCTGTC-3’), 0.24uM reverse primer (5’-TTCAGCCAATCACCTCAGTG-3′), 0.24uM SYTO9, 1X DNA polymerase GoBuffer (Promega, Wisconsin USA), 1 unit GoTaq DNA Polymerase (Promega, Wisconsin USA) and 1 ng template DNA. The PCR thermal conditions were 95 °C for 120 s; 40 cycles of: 94 °C for 30s, 60 °C for 30s, 72 °C for 45 s followed by 95 °C for 300 s, 50 °C for 120 s and high resolution melt from 75 °C to 87 °C ramping by 0.2 °C / step) and consequent high resolution melt profile analysis. High resolution melt profile was confirmed using Sanger sequencing (Forward primer: 5’ TCTGCTCCTATTGGCTGGAT3’, Reverse primer: 5’ CCCTCCGTACCAGTTCTCAA3’).

### Statistical analysis

Statistical analysis was carried out in GraphPad Prism 7. For categorical variables, a χ^2^ test was used for contingencies >2 × 2, with Fishers Exact test used for 2 × 2 contingencies. For percentage of methylated reference comparisons, a Mann-Whittey-U test was used. The null hypothesis was rejected at *p* < 0.05.

### Ethical approval

The study was approved by the QIMR Berghofer Medical Research Institute Human Research Ethics Committee and the Royal Brisbane and Women’s Hospital Ethics Committee. All participants gave informed written consent prior to participation in this study.

## Results

### Clinicopathological features

**Fig. 1:**
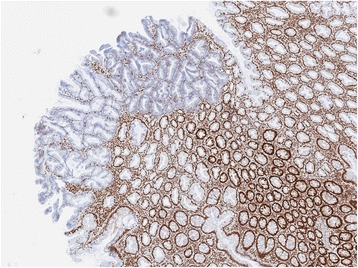
MLH1 immunohistochemistry for a sessile serrated adenoma with a focus of dysplasia. The dysplastic portion of the SSA (left) has marked loss of nuclear MLH1 expression, in contrast to the remainder of the lesion where MLH1 expression is retained

In total, there were 124 participants with SSAD, 128 with TSA, 203 with cancer and 147 controls. In accordance with study design, all polyps and cancers had the *BRAF*^*V600E*^ mutation. The allele frequency within the control cohort was similar to previously reported frequencies (22.8% vs 32.05, and 21.9% for the 1000Genomes, and TOPMED cohorts, respectively). As expected, SSADs were associated with older age, and female gender (Table 1). Immunohistochemistry for MLH1 protein demonstrated loss of expression in 75.8% of SSADs but in only one of 128 TSAs. Fig. 1 is an example of a dysplastic sessile serrated adenoma with loss of MLH1 expression isolated to the dysplastic portion of the lesion. 57.1% of BRAF mutant cancers showed loss of MLH1. The majority of all samples showed a high level of CIMP though it was less in TSAs and mismatch proficient cancers retaining MLH1 expression.

**Table 1:** Clinicopathological features

	SSAD		TSA		Cancer	
Mismatch Repair Status defined by MLH1 loss	Deficient	Proficient	Deficient	Proficient	Deficient	Proficient
Total Samples (*n*)	94	30	1	127	116	87
Mean age (years)	76.5	70.7	54.0	64.5	75.2	71.0
Male Gender	30.8%	60.0%	0%	51.1%	43.8%	69.2%
CIMP High	96.8%	86.7%	0%	59.8%	80.0%	64.7%

### MLH1–93 AA genotype associated with MLH1 protein loss in dysplastic sessile serrated adenomas and BRAF mutant cancers

We stratified SSADs according to their MLH1 protein expression and compared the frequency of each genotype (GG, GA, AA) at *MLH1*–93 (Table 2). The AA genotype was significantly more common in patients with SSADs in which there was loss of MLH1 expression, compared to control patients (SSAD with MLH1 loss versus Control, *P* = 0.037). We did not observe any instances of the AA genotype in SSADs that retained MLH1 expression. Overall, there was a significantly higher A allele frequency in SSADs with loss of MLH1 than in SSADs that retained expression (33.5% vs 15.0%, *p* < 0.01). We considered that sidedness of the dysplastic SSA may influence methylation of *MLH1*. While proximal polyps were more likely to have *MLH1* methylation and loss (*P* = 0.013), there was no association between sidedness and genotypic frequency).

**Table 2:** *MLH1*–93 single nucleotide polymorphism genotypes in controls, sessile serrated adenomas with dysplasia, traditional serrated adenomas and *BRAF* mutant cancers

	Mismatch Repair Status	Total *n*	GG *n* (%)	*P*-Value^*^	GA *n* (%)	P-Value^*^	AA *n* (%)	P-Value^*^
Controls		147	87 (59%)		53 (36%)		7 (5%)	
SSAD	Deficient	94	44 (47%)	*0.036*	37 (39%)	0.393	13 (14%)	*0.037*
	Proficient	30	21 (70%)		9 (30%)		0	
TSA	Deficient	1	0		1		0	
	Proficient	127	76 (60%)		45 (35%)		6 (5%)	
Cancer	Deficient	116	52 (44.8%)	*0.011*	51 (43.9%)	0.194	13 (11.2%)	*0.015*
	Proficient	87	55 (63.2%)		30 (34.5%)		2 (2.3%)	

*Fisher’s Exact test, significant *P*-values in italics

For colorectal cancers with MLH1 loss we observed significantly more instances of the AA genotype (11.2% vs 2.3%, *p* = 0.015) (Table 2). The genotypic frequencies of MLH1 retained *BRAF* mutant colorectal cancers was not significantly different from the control cohort. In contrast, *BRAF* mutant colorectal cancers with loss of MLH1 were more likely to harbor the A allele (*P* = 0.010). We did not observe any association between sidedness or genotype in the cancer cohort.

### Traditional serrated adenomas may harbor the AA genotype, but retain MLH1 protein expression

Traditional serrated adenomas displayed the AA genotype in 5% of cases (6/127). Strikingly, the genotypic frequency was nearly identical to that of our control cohort (Table 2). The one TSA that had loss of MLH1 had a GA genotype, and had a PMR of 140 at the *MLH1* locus, indicating that loss of MLH1 in this context is likely a result of promoter hypermethylation. We observed no relationship between sidedness and *MLH1* methylation or protein expression loss, nor was genotype significantly different when comparing locations.

### The a allele at MLH1–93 is associated with dose dependent increase in MLH1 methylation in dysplastic sessile serrated adenomas and BRAF mutant colorectal cancers.

**Fig. 2:**
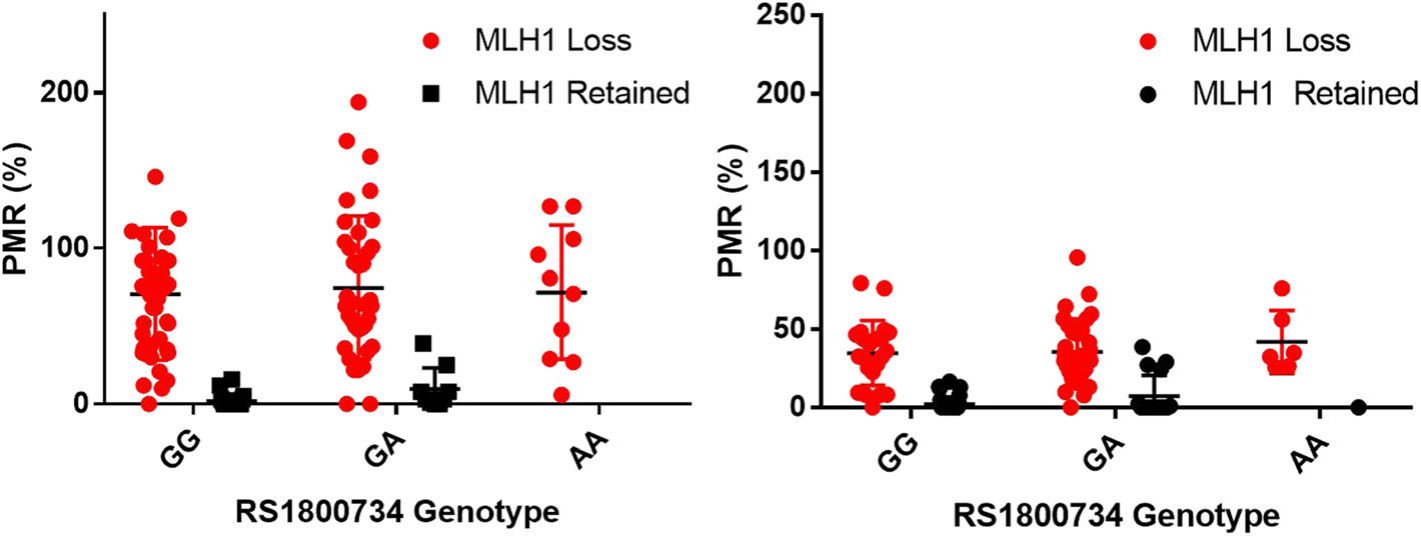
Percentage of methylated reference as per [20] (Bettington et al. 2017), of *BRAF* mutant dysplastic sessile serrated adenomas (Left) and *BRAF* mutant colorectal cancers (Right) stratified for *MLH1*–93 genotype, and MLH1 protein immunostaining

To determine whether the loss of MLH1 protein expression associated with the A allele was a result of *MLH1* promoter hypermethylation, we carried out methylation specific qPCR in all SSADs and *BRAF* mutant colorectal cancers. The A allele was associated with a significant, dose-dependent increase in the average *MLH1* promoter methylation percentage of methylated reference (PMR) value in both dysplastic SSAs (PMR 48% in GG, 62% in GA genotype and 86% in AA genotype, ANOVA, *p* = 0.022,) and *BRAF* mutant cancers (PMR 14% in GG, 23% in GA and 36% in AA, ANOVA, *p* = 0.019, Fig. 2).

## Discussion

Sessile serrated adenomas progress to malignancy following the development of focal dysplasia [10]. Approximately 75% of dysplastic SSA develop hypermethylation at *MLH1*, lose mismatch repair function and develop the MSI phenotype, whilst the rest remain mismatch repair proficient [10]. Factors involved in this bifurcation are currently unknown. The present study provides evidence that this is influenced by an inherited predisposition to *MLH1* hypermethylation via a series of germline regulatory single nucleotide polymorphisms. Our data indicates a significant increase in the A-allele at *MLH1*–93 in *BRAF* mutant, mismatch repair deficient, dysplastic sessile serrated adenomas and colorectal cancers. Further, we demonstrate a dose-dependent increase in promoter localized CpG island hypermethylation in the presence of A-alleles in the cellular context of dysplastic sessile serrated adenoma.

Intriguingly, we observed similar allele and genotype frequencies in our traditional serrated adenoma cohort as are present in our local control cohort. Traditional serrated adenomas are nearly universally microsatellite stable lesions [18]. Our data indicates that while the *MLH1*–93 A allele predisposes sessile serrated adenomas to *MLH1* hypermethylation and mismatch repair deficiency, this is not the case for traditional serrated adenomas. Instead, we propose that traditional serrated adenomas arise through distinct molecular pathways that will not, regardless of regulatory genetic changes, methylate the *MLH1* promoter. This is despite the presence of the *BRAF*^*V600E*^ mutation and CIMP. It is possible that there are quantitative and qualitative differences in CIMP and interestingly less TSAs and *BRAF* mutant, mismatch repair proficient cancers met the definition of a high level of CIMP. We postulate that *BRAF* mutant MSS colorectal cancers with the AA-genotype arise in traditional serrated adenomas.

The mechanism by which the A-allele promotes, or the G-allele prevents, methylation is unclear. Perera and colleagues [19] used EMSA assays to demonstrated the modulation of the binding of nuclear proteins to the region by the MLH1–93 G > A SNP. We and other groups [12, 13] have used bioinformatics approaches to estimate the effects of the polymorphism on transcription factor binding, identifying numerous candidate protein binding events, including the destruction of TFAP4, Pbx1b and Myf-5 binding sites and creation of AP-3, HNF-3b and GCR binding sites in the presence of the A-allele. Savio and colleagues [13] used ChIP assays to demonstrate the diminished binding of TFAP4 in cell lines of AA-genotype confirming the accuracy of at least one of our predictions. Interestingly, TFAP4 is under-expressed in CIMP-positive cancers. TFAP4 may share similar affinity for specific sequences as the protein complexes involved in maintenance of CIMP, and hence could be repressed in order to promote the CIMP phenotype.

The loss of mismatch repair function and development of MSI within sessile serrated adenomas with dysplasia is highly clinically relevant as these lesions evolve rapidly into invasive cancer, often in less than 12 months [10]. *BRAF* mutant MSI colorectal cancers have an excellent 5 year survival of 84.6%, while microsatellite stable *BRAF* mutant colorectal cancers have a significantly reduced 5-year survival of 40.5% [9]. There is no evidence that the *MLH1*–93 polymorphism makes an individual more likely to develop sessile serrated adenomas but if they do, the present study suggests the outcome is likely to be better if they carry the A allele, especially if they are homozygous AA because if a cancer develops it is likely to be MSI. However, other factors must also be important as a number of SSADs with loss of MLH1 expression possessed the GG genotype. These lesions may have polymorphisms in other regions of the genome modulating methylation at the locus, or possess other risk factors for *MLH1* promoter hypermethylation. Understanding other genetic and environmental risk factors that predispose a sessile serrated adenoma to MLH1 retention will aid in evaluating patients who are at risk of developing these particularly aggressive cancers, and may inform surveillance guidelines.

## Conclusion

In conclusion, inheritance of the A allele is associated with a dose dependent increase in methylation at the *MLH1* promoter in dysplastic sessile serrated adenomas. The homozygous A genotype appears to *strongly* predict the development of mismatch repair deficiency at the transition to dysplasia in this context. However, the A allele is insufficient to generate *MLH1* methylation and loss of protein expression in other cellular contexts, such as traditional serrated adenoma in the present study and in PBMCs as reported by Miyakura et al. [12].

We propose that the *MLH1* polymorphism is an important risk factor for development of *MLH1* methylation but only in certain cellular environments such as sessile serrated adenomas and *BRAF* mutant colorectal cancers arising from sessile serrated adenomas. Collectively, these findings inform our understanding of the mechanism by which *MLH1* methylation can occur in the setting of serrated colorectal neoplasia. Understanding the implications of germline polymorphisms in the epigenetic modulation of gene expression may inform screening guidelines and risk stratification for patients with sessile serrated adenomas.

## Abbreviations

CIMP: CpG Island Methylator Phenotype
MSI: Microsatellite Unstable/Instability
MSS: Microsatellite stable
SSA: Sessile serrated adenoma
SSAD: Dysplastic sessile serrated adenoma
TSA: Traditional serrated adenoma
TSAD: Dysplastic traditional serrated adenoma
